# Prognostic value of laboratory biomarkers for mortality risk stratification in thrombotic thrombocytopenic purpura

**DOI:** 10.1007/s00277-025-06584-8

**Published:** 2025-08-30

**Authors:** Xu Xiang, Yue-qing Dai

**Affiliations:** 1 Department of Laboratory Medicine, Tongji Hospital, Tongji Medical College, Huazhong University of Science and Technology, Wuhan, 430030 China; 2 Department of Gastroenterology, Tongji Hospital, Tongji Medical College, Huazhong University of Science and Technology, 1095 Jiefang Ave, Wuhan, 430030 China

**Keywords:** Thrombotic thrombocytopenic purpura, Cardiac troponin I, Lactate dehydrogenase, Mortality risk, Risk stratification model

## Abstract

This study aimed to explore the relationship between laboratory indicators and short-term mortality risk in patients with thrombotic thrombocytopenic purpura (TTP) and to construct a risk stratification model. We retrospectively analyzed the clinical data of 106 patients with TTP admitted to Tongji Hospital between June 2016 and February 2025. Patients were grouped by 28-day survival: death (*n* = 45) and survival (*n* = 61). Prognosis-related indicators were identified using receiver operating characteristic (ROC) curves and logistic regression analyses. A mortality risk stratification model was established. To validate the stability of the model, 30 external cases of TTP (January 2022–December 2024) were collected. The levels of cardiac troponin I (cTnI), lactate dehydrogenase (LDH), blood urea nitrogen (BUN), and indirect bilirubin (IBIL) were significantly higher in the death group compared to those in the survival group. ROC analysis identified optimal mortality risk cutoffs: cTnI at 353.1 pg/mL (odds ratio [OR] = 4.778), LDH at 992 U/L (OR = 2.842), BUN at 10.9 mmol/L (OR = 5.527), and IBIL at 32.3 µmol/L (OR = 2.995). A subsequent 0–4 point risk stratification model demonstrated increasing mortality rates of 21.3% (0–1 point), 39.1% (2 points), 60.9% (3 points), and 92.3% (4 points). Each 1-point increase in the risk score was associated with a 2.324-fold rise in mortality risk (95% confidence interval 1.597–3.383). Internal and external validations confirmed the model’s stability and strong prognostic performance in patients with TTP. The risk model incorporating cTnI, LDH, BUN, and IBIL levels effectively predicted short-term mortality in patients with TTP. Patients with a score of 3 or higher required close monitoring and aggressive therapeutic intervention to mitigate mortality risk.

**Supplementary Information:**

The online version contains supplementary material available at 10.1007/s00277-025-06584-8.

## Introduction

Thrombotic thrombocytopenic purpura (TTP) is a rare, rapidly progressive hematologic emergency characterized by severe thrombocytopenia, microangiopathic hemolytic anemia, and multi-organ ischemic injury [1]. The core pathogenesis of TTP is closely linked to a severe von Willebrand factor-cleaving protease 13 (ADAMTS13) deficiency. Based on etiology, TTP can be classified as hereditary (congenital TTP, cTTP) or immune-mediated (acquired TTP, iTTP). cTTP arises from mutations in the ADAMTS13 gene, causing congenital enzyme deficiency. Although the condition accounts for approximately 5% of clinical cases, its prevalence increases to 25–50% in pediatric and pregnant populations. In contrast, iTTP results from anti-ADAMTS13 autoantibody-mediated acquired enzyme inhibition or accelerated clearance, and represents approximately 95% of all cases [2]. Laboratory confirmation of TTP typically demonstrates thrombocytopenia, hemolytic anemia, elevated lactate dehydrogenase (LDH) levels, and profoundly reduced ADAMTS13 activity (< 10%) [3]. Timely diagnosis of life-threatening TTP, essential for reducing its high mortality rate, remains a significant clinical challenge. Beyond diagnosis, prognostic stratification through mortality risk assessment assumes paramount importance in guiding therapeutic intensity, particularly in identifying patients who may benefit from early adjunctive therapies such as rituximab or caplacizumab, while avoiding potentially harmful platelet (PLT) transfusions in confirmed cases [2]. This retrospective cohort study of 106 patients with TTP systematically evaluated prognostic laboratory parameters and developed a novel mortality risk stratification model to guide clinical decision making, ultimately aiming to optimize patient outcomes through evidence-based risk-adapted management strategies.

## Methods

### Criteria for patient inclusion and exclusion

This retrospective analysis included 106 consecutive patients with TTP treated at Tongji Hospital, Tongji Medical College, Huazhong University of Science and Technology (Wuhan, China) between June 2016 and February 2025. The cohort comprised 45 males and 61 females, with a mean age of 48.631 ± 18.393 years. Moreover, all patients underwent ADAMTS-13 activity testing. Twenty-three patients received rituximab in combination with standard treatment (plasma exchange [PEX] + glucocorticoids), while the remainder received standard treatment alone. Diagnosis required fulfillment of international criteria for TTP: (1) plasma ADAMTS13 activity < 10%, or (2) ADAMTS13 activity < 20% with concurrent ADAMTS13 inhibitor positivity or confirmed genetic mutations. Exclusion criteria comprised: (1) incomplete laboratory documentation; (2) cases diagnosed as outpatients without hospitalization; and (3) concurrent hematologic malignancies. The Institutional Review Board of the study center approved this protocol.

### Data collection and categorization

This retrospective case-control study employed consecutive enrollment to comprehensively analyze demographic characteristics, clinical presentations, and laboratory parameters. Data collection encompassed: (1) baseline variables (age, gender, temperature, alteration of consciousness, days of hospitalization, days from onset to admission, treatment modality); (2) admission laboratory profiles included levels of white blood cell (WBC), neutrophil (NEUT), lymphocyte (LYM), red blood cell (RBC), hemoglobin (HGB), PLT, as well as neutrophil-to-lymphocyte ratio (NLR), red cell distribution width (RDW), activated partial thromboplastin time (APTT), prothrombin time (PT), thrombin time (TT), D-dimer (DD), fibrinogen (FIB), cardiac troponin I (cTnI), alanine aminotransferase (ALT), aspartate aminotransferase (AST), total proteins (TP), albumin (ALB), globulin (GLB), total bilirubin (TB), direct bilirubin (DB), indirect bilirubin (IBIL), alkaline phosphatase (ALP), gamma-glutamyl transpeptidase (GGT), LDH, creatinine (CREA), blood urea nitrogen (BUN), uric acid (UA), potassium (K), sodium (NA), chloride (CL), calcium (CA), bicarbonate (HCO3), and ADAMTS13 activity and inhibitor analysis. The 28-day post-admission period served as the primary observational endpoint, with patients stratified into death and survival cohorts based on outcomes.

### Instruments and reagents

Measurement consistency was ensured using standardized platforms: hematologic parameters via Sysmex XE-2100; coagulation studies with STAGO analyzer and manufacturer reagents; cardiac biomarkers using ARCHITECT i2000SR immunoassay; and biochemical profiles analyzed by Roche Cobas c8000. Plasma ADAMTS13 activity was assessed using the fluorescence resonance energy transfer substrate assay (Sekisui Diagnostics, Stamford, CT, USA). Inhibitory antibodies were detected by incubating patient plasma with normal pooled plasma (1:1) at 37 °C for 2 h; residual activity below 50% of control indicated significant inhibition. Genetic testing for hereditary TTP was performed in patients with undetectable inhibitors via Sanger sequencing of the ADAMTS13 exons.

### Statistical methods

Statistical analyses were performed using SPSS (version 20.0; IBM Corp.), and data normality was evaluated using the Shapiro–Wilk test. Continuous variables were expressed as mean ± standard deviation for normally distributed data, or median [P25, P75] for non-normal distributions. Group comparisons employed independent samples t-tests for normally distributed variables, Mann–Whitney U tests for skewed data, and χ² tests for categorical proportions. Receiver operating characteristic (ROC) curve analysis identified optimal mortality cutoffs, with sensitivity, specificity, and area under the curve (AUC) calculated to assess prognostic performance. Multivariable logistic regression was employed to quantify the mortality risk using odds ratios (OR) and 95% confidence intervals (CI). Statistical significance was set at *p* < 0.05.

## Results

### Demographic and clinical characteristics of patients with TTP

The differences between the two groups in terms of sex, temperature at admission, and days from onset to admission were not statistically significant (*p* > 0.05). However, the survival group had a lower age and fewer cases with altered consciousness, but longer hospital stays compared to those in the death group. In addition, the proportion of patients receiving rituximab combined with standard treatment was significantly higher in the survival group than in the death group (*p* < 0.05). See Table 1.

**Table 1:** Comparison of demographic and clinical characteristics between the survival group and the death group in TTP patients

	Survival group (*n* = 61)	Death group (*n* = 45)	t/z/χ² value	*P*-value
Age (years)	43.49 ± 19.35	55.60 ± 14.31	−3.668	< 0.001
Gender [n(%)]				
male	24 (39.34)	21 (46.6)	0.568	0.451
female	37 (60.65)	24 (53.33)		
Admission temperature (℃)	36.60[36.50, 37.00]	36.60[36.40, 36.90]	0.681	0.495
Alteration of consciousness	22 (36.06)	29 (64.44)	8.354	0.004
Days of hospitalization	16.00[10.00, 22.00]	4.00 [2.00,6.00]	7.622	< 0.001
Days from onset to admission	5.00 [3.00,10.00]	6.00[3.00,7.00]	0.502	0.615
Treatment modality [n(%)]				
Rituximab combined with standard treatment	20(32.79)	3 (6.67)	10.399	0.001
plasma exchange + glucocorticoid	41(67.21)	42(93.33)		

### Comparison of baseline characteristics between survival and death groups

No statistically significant differences were noted between groups in WBC, NEU, LYM, NLR, RDW, RBC, HGB, PLT, APTT, PT, TT, FIB, D-D, AST, ALT, TP, ALB, ALP, GGT, UA, K, NA, CL, CA, HCO3, and ADAMTS13 activities (*p* > 0.05). In the death group, levels of cTnI, TB, DB, IBIL, LDH, BUN, and CREA were significantly elevated compared to the levels observed in the survival group. Moreover, the differences were statistically significant (*p* < 0.05). See Table 2.

**Table 2:** Comparison of baseline characteristics between the survival group and the death group in TTP patients

	Survival group (*n* = 61)	Death group (*n* = 45)	t/z/χ² value	*P*-value
WBC (10^9^/L)	7.88[5.60, 11.40]	7.54 [6.15, 11.34]	−0.058	0.957
RBC (10^12^/L)	2.27 ± 0.69	2.50 ± 0.62	−1.685	0.095
LYM (10^9^/L)	1.19 [0.86,1.81]	1.11 [0.77,1.65]	1.477	0.141
HGB(g/L)	70.29 ± 19.82	75.51 ± 17.00	−1.407	0.162
PLT (10^9^/L)	10.00 [6.00, 15.00]	8.00 [5.00,13.00]	1.361	0.173
NEUT (10^9^/L)	5.54 [3.93,8.20]	5.80 [4.56, 7.98]	−0.687	0.494
NLR	3.96 [2.12, 8.15]	5.04 [3.54,8.09]	−1.697	0.090
RDW (%)	18.60 [15.60, 21.90]	16.60 [14.90, 23.10]	0.866	0.388
APTT (S)	37.20 [34.20, 43.00]	38.50 [35.70, 42.10]	−0.633	0.529
PT(S)	14.30 [13.50, 15.30]	15.00 [14.10, 15.90]	−1.796	0.073
TT (S)	17.30 [16.30, 18.10]	17.40 [16.60, 19.00]	−0.924	0.357
DD (µg/mL)	2.79 [1.49,5.40]	3.14 [2.03,7.71]	−0.965	0.336
FIB (g/L)	3.26 [2.61,4.04]	3.20 [2.48, 4.13]	−0.169	0.868
cTnI (pg/ml)	164.52 [32.60,539.50]	712.10[291.80, 3077.90]	−3.774	< 0.001
ALT (U/L)	17.00 [12.00, 27.00]	21.00[13.00, 31.00]	−1.304	0.193
AST (U/L)	40.92 [30.00, 64.00]	49.00 [37.00, 76.00]	−1.774	0.077
TP (g/L)	66.88 ± 6.37	64.69 ± 7.87	1.566	0.120
ALB (g/L)	37.50 [33.70, 40.80]	37.20 [34.20, 38.30]	1.269	0.206
GLB (g/L)	30.11 ± 5.11	29.10 ± 5.58	0.950	0.344
TB (µmol/L)	40.70 [32.70, 56.80]	56.30 [32.50, 73.80]	−2.097	0.036
DB (µmol/L)	11.50 [8.80, 14.50]	14.90 [10.10, 19.50]	−2.413	0.016
IBIL (µmol/L)	27.90 [20.50, 39.20]	40.80 [22.70, 54.30]	−1.989	0.049
ALP (U/L)	67.00 [50.00, 95.00]	63.00 [52.00, 74.00]	0.837	0.404
LDH (U/L)	990.0[841.0, 1523.0]	1224.0[992.0, 1601.0]	−2.004	0.045
GGT (U/L)	23.00 [16.00, 39.00]	24.00 [16.00, 44.00]	−0.355	0.725
BUN (mmol/L)	7.20 [5.20, 9.30]	10.90 [7.40, 17.50]	−3.416	< 0.001
CREA (µmol/L)	72.00 [55.00, 95.00]	100.00 [70.00, 140.00]	−3.621	< 0.001
UA (µmol/L)	307.00[240.00,438.00]	370.000[286.10,482.20]	−1.195	0.233
K (mmol/L)	4.05 ± 0.46	4.14 ± 0.68	−0.767	0.446
NA (mmol/L)	138.20[136.80, 139.90]	138.40[136.80, 140.20]	−0.029	0.980
CL (mmol/L)	102.52 ± 3.32	101.22 ± 3.53	1.917	0.058
CA (mmol/L)	2.13 [2.05,2.25]	2.11 [1.98, 2.20]	1.643	0.101
HCO3 (mmol/L)	22.00 [19.10, 24.50]	21.70 [19.00, 24.10]	0.374	0.711
ADAMTS13 activity (%)	5.00 [4.00,7.10]	5.00 [3.00, 6.50]	0.454	0.646

*WBC* white blood cell, *NEUT* neutrophils, *LYM* lymphocytes, *RBC* red blood cells, *HGB* hemoglobin, *PLT* platelet, *NLR* neutrophil-to-lymphocyte ratio, *RDW* red cell distribution width, *APTT* activated partial thromboplastin time, *PT* prothrombin time, *TT* thrombin time, *DD* d-dimer, *FIB* fibrinogen, *cTnI* cardiac troponin I, *ALT* alanine aminotransferase, *AST* aspartate aminotransferase, *TP* total protein, *ALB* albumin, *GLB* globulin, *TB* total bilirubin, *DB* direct bilirubin, *IBIL* indirect bilirubin, *ALP* alkaline phosphatase, *GGT* gamma-glutamyl transpeptidase, *LDH* lactate dehydrogenase, *CREA* creatinine, *BUN* blood urea nitrogen, *UA* uric acid, *K* potassium, *NA* sodium, *CL* chloride, *CA* calcium, and *HCO3* bicarbonate

### Evaluating the prognostic performance of biomarkers and developing the TTP mortality risk stratification model

**Fig. 1:**
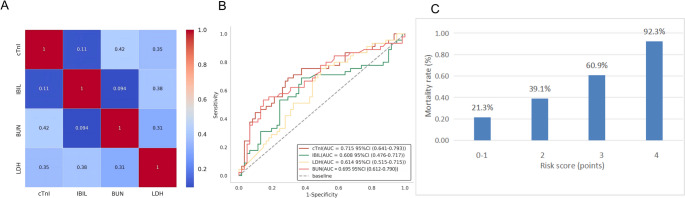
Evaluating the prognostic performance of biomarkers and developing the TTP mortality risk stratification model​​ (**A**) Correlation matrix of four candidate biomarkers. Color scale: red (strong positive correlation) to blue (weak correlation) (**B**) Receiver operating characteristic (ROC) curves for individual biomarkers predicting 28-day mortality. Optimal cutoffs and area under the curve (AUC): cTnI: 353.1 pg/mL (AUC = 0.715, 95% CI 0.632–0.816), LDH: 992 U/L (AUC = 0.614, 0.510–0.726), BUN: 10.9 mmol/L (AUC = 0.695, 0.587–0.782), IBIL: 32.3 µmol/L (AUC = 0.608, 0.480–0.730). Dashed vertical lines denote cutoff thresholds (**C**) Mortality rates stratified by risk score (0–4 points). Bars represent observed mortality: 21.3% (0–1 points), 39.1% (2 points), 60.9% (3 points), 92.3% (4 points); error bars indicate 95% confidence intervals. The solid trend line reflects logistic regression analysis (OR = 2.324 per 1-point increase, 95% CI 1.597–3.383; *p* < 0.001)

Pearson’s correlation analysis was performed on seven statistically significant variables (cTnI, TB, DB, IBIL, LDH, BUN, and CREA) to assess multicollinearity. Three parameters (TB, DB, and CREA) demonstrating pairwise correlations > 0.7 were excluded to prevent model overfitting, leaving four variables (cTnI, IBIL, LDH, and BUN) for subsequent analysis, as illustrated in Fig. 1A. The variance inflation factor (VIF) evaluation confirmed acceptable multicollinearity across all retained variables (VIF < 10), eliminating the need for further variable exclusion.ROC analysis identified the optimal cutoff values for predicting mortality in patients: cTnI at 353.1 pg/mL, LDH at 992 U/L, BUN at 10.9 mmol/L, and IBIL at 32.3 µmol/L, with corresponding AUC values of 0.715 (95% CI 0.632–0.816), 0.614 (0.510–0.726), 0.695 (0.587–0.782), and 0.608 (0.480–0.730), respectively (Fig. 1B; Table 3). Patients exceeding these cut-off values exhibited a significantly higher mortality risk. Multivariable logistic regression analysis revealed progressively increased odds of death with elevated biomarkers: cTnI > 353.1 pg/mL (OR = 4.778, 95% CI 2.086–10.944, *p* < 0.001), LDH > 992 U/L (OR = 2.842, 1.239–6.515, *P* = 0.014), BUN > 10.9 mmol/L (OR = 5.527, 2.207–13.837, *p* < 0.001), and IBIL > 32.3 µmol/L (OR = 2.995, 1.345–6.667, *p* = 0.007) (Table 4). Based on these biomarkers, a 0–4 point TTP mortality risk stratification model was developed, assigning 1 point for each biomarker exceeding its respective cutoff. The model demonstrated progressively increasing mortality rates: 21.3% (0–1 points), 39.1% (2 points), 60.9% (3 points), and 92.3% (4 points). Logistic regression analysis demonstrated that each 1-point increase in the risk score was associated with a 2.324-fold rise in mortality risk (OR = 2.324, 95% CI 1.597–3.383, *p* < 0.001), confirming the ability of the model to stratify mortality risk in patients with TTP (Fig. 1C). The TTP mortality risk score calculator developed in this study is available as an Excel template and can be accessed via the following links: Tencent Weiyun link: https://share.weiyun.com/6Kgsz3tn and Google Drive link: https://docs.google.com/spreadsheets/d/1wdLKNgHygALGQheiSpyBJWDo2n4hrkur/edit?usp=drive_link%26;ouid=101198135748562103354%26;rtpof=true%26;sd=true. The utilization of the calculator is illustrated in Supplementary Fig. 1. Upon entering the values for the four indicators (cTnI, LDH, BUN, and IBIL), the template automatically generates the total score (0–4 points), estimates the corresponding mortality risk probability, and provides relevant clinical recommendations.

**Table 3:** Diagnostic performances of cTnI, LDH, BUN and IBIL for distinguishing the death group from the survival group in TTP patients

Variable	Cut-Off value	AUC (95%CI)	Sensitivity (%)	Specificity(%)	Youden Index
cTnI(pg/ml)	353.10	0.715 (0.632–0.816)	68.9%	70.5%	0.394
LDH (U/L)	992.00	0.614 (0.510–0.726)	75.6%	50.8%	0.264
BUN(mmol/L)	10.90	0.695 (0.587–0.782)	53.3%	85.2%	0.386
IBIL(µmol/L)	32.30	0.608 (0.480–0.730)	68.9%	62.3%	0.312

*AUC* area under the curve, *cTnI* cardiac troponin I, *LDH* lactate dehydrogenase, *BUN* blood urea nitrogen, *IBIL* indirect bilirubin

**Table 4:** cTnI, LDH, BUN and IBIL predict short-term risk of death in TTP patients

Variable		OR value	95% CI	*P*-value
cTnI_Group	<353.1			
	>353.1	4.778	[2.086,10.944]	< 0.001
LDH_Group	<992.0			
	>992.0	2.842	[1.239,6.515]	0.014
BUN_Group	<10.90			
	>10.90	5.527	[2.207,13.837]	< 0.001
IBIL_Group	<32.30			
	>32.30	2.995	[1.345,6.667]	0.007

*OR* odds ratios, *CI* confidence intervals, *cTnI* cardiac troponin I, *LDH* lactate dehydrogenase, *BUN* blood urea nitrogen, *IBIL* indirect bilirubin

### Internal and external validation of the model

**Fig. 2:**
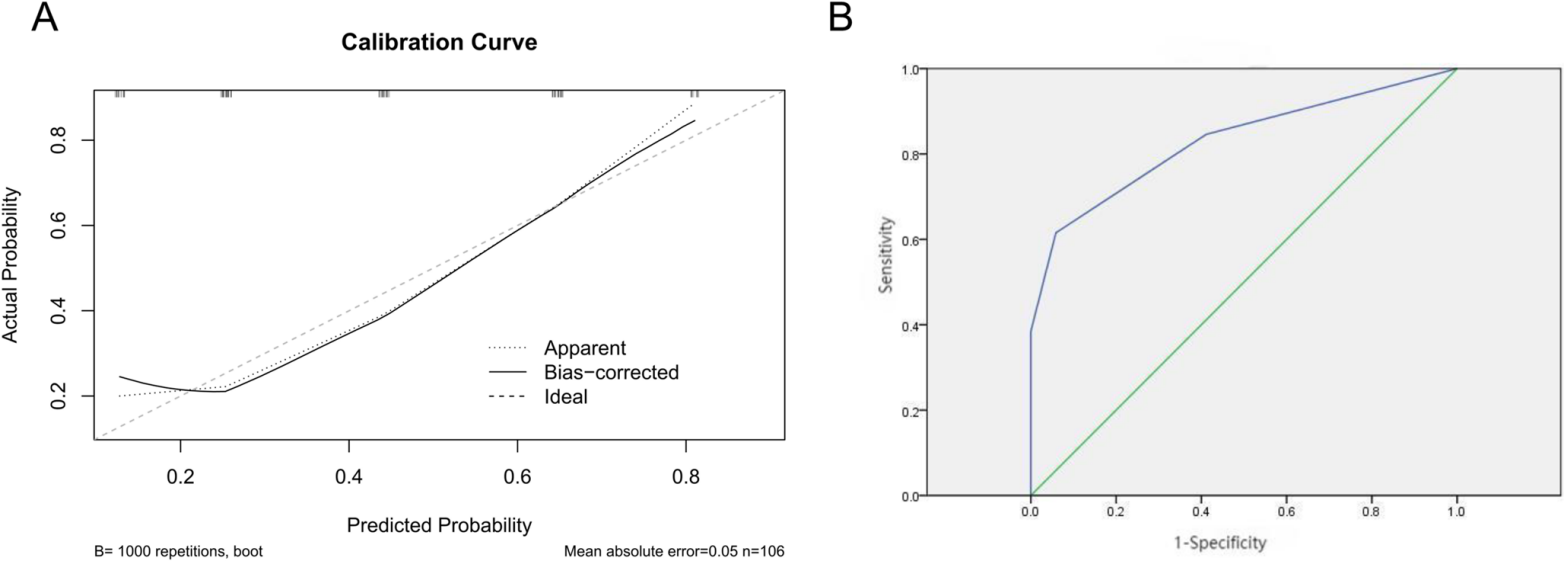
Validation of the TTP mortality risk stratification model​​ (**A**) Internal validation: Calibration curve from 1,000 bootstrap resamples. Diagonal dashed line indicates perfect prediction. The solid line shows agreement between predicted mortality probability and observed outcomes (mean absolute error = 0.05). Shaded area: 95% confidence band (**B**) External validation: ROC analysis of the risk model in an independent cohort (*n* = 30). AUC = 0.830 (95% CI 0.682–0.978), sensitivity = 61.5%, specificity = 94.1%, accuracy = 80%. Dotted diagonal line: reference for random prediction (AUC = 0.5)

Internal and external validations were performed to evaluate the stability and accuracy of the model. For internal validation, a bootstrap resampling method (1,000 iterations) was employed. The calibration curve closely followed the diagonal line, indicating good agreement between predicted probabilities and observed outcomes, with an average absolute error of 0.05 (Fig. 2A). For external validation, the model was applied to 30 patients with TTP from an independent cohort. The levels of cTnI, LDH, BUN, and IBIL were integrated into the pre-established TTP mortality risk stratification model, and the predicted and actual risks were compared (Table 5). This model exhibited a robust predictive performance, with an AUC of 0.830, sensitivity of 61.5%, specificity of 94.1%, and accuracy of 80% (Fig. 2B).

**Table 5:** External validation of the TTP mortality risk stratification model

Score	Predicted Risk (%)	Actual Risk (%)	Number of External Validation patients	Number of Deaths
0–1	21.3	16.7	12	2
2	39.1	33.3	9	3
3	60.9	75.0	4	3
4	92.3	100.0	5	5

## Discussion

TTP is a highly aggressive condition with a mortality rate reaching up to 90% if left untreated. Moreover, clinical manifestations often overlap with other thrombotic microangiopathies (e.g., hemolytic uremic syndrome), which complicates early diagnosis [4]. Currently, the first-line treatment for TTP is PEX, which significantly reduces its associated mortality. In clinical practice, glucocorticoids are typically empirically used in combination with PEX. Glucocorticoids are believed to assist PEX in inhibiting antibodies and inflammatory responses, protecting organ function, and reducing inhibitor titers [5]. In addition to early initiation of PEX, studies have demonstrated that combining rituximab with refractory TTP can accelerate disease remission and effectively prevent recurrence [6]. In recent years, with the application of caplacizumab, the mortality rate of TTP has significantly reduced to less than 2% [7]. However, early identification of high-risk patients remains crucial for further optimization of treatment. Previous research indicates that dynamic changes in ADAMTS13 activity and antibody levels not only underpin disease classification but also closely correlate with recurrence risk and prognosis [8]. Additionally, laboratory indicators such as PLT count and elevated LDH and IBIL levels may reflect the degree of microvascular hemolysis and organ damage progression, although their quantitative associations with mortality risk remain unclear.

In this study, we analyzed laboratory indices of 106 patients with TTP and identified four significant laboratory markers (cTnI, LDH, BUN, and IBIL) strongly associated with short-term mortality risk. Based on these findings, we developed a TTP mortality risk stratification model integrating multiple indices, offering a novel approach for clinical prognostic assessment. Patients in the mortality group exhibited significantly elevated cTnI, BUN, LDH, and IBIL levels, suggesting that cumulative organ damage plays a critical role in determining prognosis.

Elevated levels of cTnI, a marker of myocardial injury, are directly linked to microvascular thrombosis-induced myocardial necrosis. A study by the French Reference Center for Thrombotic Microangiopathy demonstrated that an initial cTnI level > 0.25 µg/L increased the risk of death or refractory TTP threefold [9]. However, the cutoff value in our study was high (353.1 pg/mL), potentially reflecting differences in the assay methodology or population characteristics. Notably, acute myocardial infarction as a complication of TTP has been independently associated with increased in-hospital mortality [10], consistent with the prognostic significance of cTnI.

Renal impairment is frequently observed in patients with TTP, which results in decreased BUN excretion and elevated BUN levels. Renal insufficiency may exacerbate microvascular thrombosis, further impacting the prognosis. Furthermore, as an autoimmune disease, TTP induces inflammatory responses that increase protein catabolism, leading to elevated BUN production. Inflammation may also impair renal function and hinder the excretion of BUN. Additionally, high BUN levels may indicate a catabolic state or malnutrition, both of which are associated with poor outcomes. High BUN levels may also reflect oxidative stress and inflammatory responses, which can impair the vascular endothelial function and exacerbate TTP.

LDH serves as a marker of hemolysis and tissue ischemia, primarily reflecting erythrocyte destruction and release from damaged tissues. Dynamic changes in LDH levels sensitively reflect therapeutic responses, with persistent elevation indicating refractory thrombosis that may necessitate adjustment of PEX protocols or the addition of complement inhibitors [11].

Increased IBIL levels result from the accumulation of hemoglobin metabolites following erythrocyte destruction. Studies have confirmed that combining IBIL with LDH significantly enhances the diagnostic specificity of TTP, particularly when ADAMTS13 detection is limited [12]. Notably, ADAMTS13 activity did not differ significantly between groups, suggesting that enzyme deficiency alone does not drive acute-phase mortality. Instead, “second-strike” mechanisms of organ damage (e.g., inflammation or superimposed endothelial injury) may play a more critical role [8].

The 0–4-point risk scoring system developed in this study demonstrated that patients with a score ≥ 3 exhibited an alarmingly high mortality rate of 60.9–92.3%, significantly surpassing that of the low-risk group (21.3%). This model holds several important clinical implications: First, elevated LDH (> 992 U/L) or IBIL (> 32.3 µmol/L) levels can serve as early warning indicators of occult organ damage, warranting close monitoring even when PLT counts have not reached critical thresholds. Second, high-risk patients need early intensive therapy, such as the combination of rituximab or caplacizumab. In this study, the mortality rate in the group receiving rituximab combined with standard treatment was significantly lower than that of the standard treatment group alone, further supporting the need to add rituximab or caplacizumab to the PEX + glucocorticoids for high-risk patients (score ≥ 3) to rapidly inhibit microthrombosis formation. Low-risk patients (0–1 score) may avoid overtreatment (such as prolonging the PEX course), but still require close monitoring of dynamic changes in clinical indicators [13]. The mortality risk stratification model provides an evidence-based basis for the precise use of rituximab and helps optimize the allocation of medical resources. Dynamic monitoring of LDH and IBIL levels facilitates the assessment of therapeutic response, with persistently elevated values indicating the need to adjust PEX frequency or explore second-line treatment options [14, 15].

The limitations of this study include its retrospective design, which may introduce selection bias; lack of dynamic monitoring of marker changes concerning treatment response; and sample size constraints for analyzing rare complications. Future studies should be designed as multicenter prospective investigations incorporating ultra-large von Willebrand factor multimers and complement activation markers (e.g., C5b–9) to further enhance the accuracy and comprehensiveness of the risk stratification model. Additionally, exploring novel markers, including immature PLT count in conjunction with conventional indices, may enhance diagnostic and prognostic accuracy.

In conclusion, the combined detection of cTnI, LDH, BUN, and IBIL is a robust tool for identifying high-risk patients with TTP. Admission values of LDH > 992 U/L, IBIL > 32.3 µmol/L, cTnI > 353.1 pg/mL, and BUN > 10.9 mmol/L indicate a markedly elevated risk of death, underscoring the necessity for multidisciplinary collaborative care and personalized treatment adjustments. This study underscores the critical role of organ damage monitoring in TTP management and provides an evidence-based foundation for improving patient outcomes.

## Supplementary Information

Below is the link to the electronic supplementary material.

Supplementary Material 1 (PDF 291 KB)

Supplementary Material 2 (JPG 72.9 KB)

## Data Availability

The datasets supporting the findings of this study are obtainable from the corresponding author upon reasonable request.
