# Supplementary material for: Prognostic value of laboratory biomarkers for mortality risk stratification in thrombotic thrombocytopenic purpura
Source: Ann Hematol. 2025 Aug 30;104(9):4457–64. doi: 10.1007/s00277-025-06584-8 (PMC12552283; doi:10.1007/s00277-025-06584-8)
Supplement: Supplementary file 1 — Supplementary Material 1 (PDF 291 KB) [file 277_2025_6584_MOESM1_ESM.pdf]

editage

Editing Certificate

This document certifies that the manuscript listed below has been edited to ensure language and grammar accuracy and is error free in these aspects. The edit was performed by professional editors at Editage, a brand of Cactus Communications. The author's core research ideas were not altered in any way during the editing process. The quality of the edit has been guaranteed, with the assumption that our suggested changes have been accepted and the text has not been further altered without the knowledge of our editors.

MANUSCRIPT TITLE

Prognostic Value of Laboratory Biomarkers for Mortality Risk Stratification in Thrombotic Thrombocytopenic Purpura

AUTHORS

Xu Xiang , Yue-qing Dai

ISSUED ON

June 26, 2025

JOB CODE

FMXUH_2

THE EDITAGE Promise QUALITY GUARANTEE

Prabh Grewal
Senior Vice President - Editage

editage | helping you get published

Since 2002, Editage has helped over 430,000 authors publish around 1.2 million research papers in scholarly journals across over 1000 disciplines through editorial, translation, transcription, and publication support services. Editage is a brand of Cactus Communications (cactusglobal.com), a science communication and technology company.

CACTUS.

GLOBAL :
+1(669) 272-1214 | request@editage.com

CHINA :
400-001-8237; 021-60209400 | fabiao@editage.cn

editage.com | editage.co.kr | editage.jp | editage.cn | editage.com.br | editage.com.tw | editage.de
