# Supplementary figures and images for: Prognostic value of laboratory biomarkers for mortality risk stratification in thrombotic thrombocytopenic purpura
Source: Ann Hematol. 2025 Aug 30;104(9):4457–64. doi: 10.1007/s00277-025-06584-8 (PMC12552283; doi:10.1007/s00277-025-06584-8)

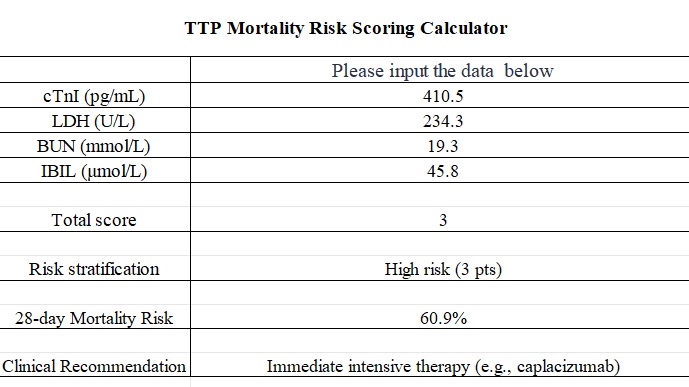

Supplement: Supplementary file 2 — Supplementary Material 2 (JPG 72.9 KB) [file 277_2025_6584_MOESM2_ESM.jpg]
